# Exogenous l-proline improved *Rhodosporidium toruloides* lipid production on crude glycerol

**DOI:** 10.1186/s13068-020-01798-6

**Published:** 2020-09-14

**Authors:** Rasool Kamal, Yuxue Liu, Qiang Li, Qitian Huang, Qian Wang, Xue Yu, Zongbao Kent Zhao

**Affiliations:** 1 Laboratory of Biotechnology, Dalian Institute of Chemical Physics, CAS, 457 Zhongshan Road, Dalian, 116023 People’s Republic of China; 2 Dalian Key Laboratory of Energy Biotechnology, Dalian Institute of Chemical Physics, CAS, 457 Zhongshan Road, Dalian, 116023 People’s Republic of China; 3 University of Chinese Academy of Sciences, Beijing, 100049 People’s Republic of China

**Keywords:** Anti-stress agent, Crude glycerol, Microbial lipids, *Rhodosporidium toruloides*, Two- stage culture

## Abstract

**Background:**

Crude glycerol as a promising feedstock for microbial lipid production contains several impurities that make it toxic stress inducer at high amount. Under stress conditions, microorganisms can accumulate l-proline as a safeguard. Herein, l-proline was assessed as an anti-stress agent in crude glycerol media.

**Results:**

Crude glycerol was converted to microbial lipids by the oleaginous yeast *Rhodosporidium toruloides* CGMCC 2.1389 in a two-staged culture mode. The media was supplied with exogenous l-proline to improve lipid production efficiency in high crude glycerol stress. An optimal amount of 0.5 g/L l-proline increased lipid titer and lipid yield by 34% and 28%, respectively. The lipid titer of 12.2 g/L and lipid content of 64.5% with a highest lipid yield of 0.26 g/g were achieved with l-proline addition, which were far higher than those of the control, i.e., lipid titer of 9.1 g/L, lipid content of 58% and lipid yield of 0.21 g/g. Similarly, l-proline also improved cell growth and glycerol consumption. Moreover, fatty acid compositional profiles of the lipid products was found suitable as a potential feedstock for biodiesel production.

**Conclusion:**

Our study suggested that exogenous l-proline improved cell growth and lipid production on crude glycerol by *R. toruloides*. The fact that higher lipid yield as well as glycerol consumption indicated that l-proline might act as a potential anti-stress agent for the oleaginous yeast strain.

## Background

Biodiesel is a renewable alternative to fossil fuels [1]. Its global production has increased dramatically in the past decade [2]. Unlike fossil fuels, biodiesel is eco-friendly and non-toxic with less sulfur and carbon dioxide emissions [3]. However, biodiesel production generates about 10% crude glycerol as a by-product, which contains several impurities at the risk of disposal [4]. As a result, several European countries treat it as industrial wastewater [5]. For this reason, the conversion of crude glycerol to value-added products is essential [1].

The current biodiesel technology is based on plant oils and animal fats, but these resources are limited. The lipid produced by oleaginous microorganisms is known to be a novel feedstock for biodiesel production with similar fatty acids composition to that as plants oil [6]. Moreover, microbial lipid has several leads over plants oil including; free of weather and land use, have high productivity, short production cycle, and easy scalability [7]. Among others, the oleaginous yeasts are considered as potential lipid producers due to their higher growth rate, adaptation to diverse substrates, and higher lipid production yields. Until now, various organic substrates including waste cooking oils [8], different biomass derived sugars [9], amino acid-rich wastes [10], and industrially produced organic wastes [11], have been utilized for microbial lipid production. However, the high production cost of microbial lipid technology prevent its broader commercialization [12], where the fermentation processes and substrate costs are significant to lipid production [13]. Thereby, in sighting cost-effective raw materials for microbial lipid technology are in need.

Previously, crude glycerol has been utilized as a carbon source for valued microbial products like citric acid [14], biopolymers [15], 1,3-propanediol [16], succinic acid [17], and microbial lipid, however, the lipid yield remained lower [18]. Many microbes are unable to utilize it efficiently [19], due to the presence of methanol, salts, organic acids, and heavy metals etc., which induce toxicity at high level [20]. On the contrary, different organisms accumulate l-proline during hostile conditions. As a compatible solute, l-proline exhibits several in vitro functions [21]; it improves protein and membrane stabilization during freezing [22], elevated temperatures [23], dehydration [21], lowers the DNA melting temperature during salinity stress [24], increases proteins solubility [25], prevents proteins aggregation [26], scavenges reactive oxygen species (ROS) [27, 28], and prevents ROS mediated cell inhibition [29, 30]. Although, the exact mechanism for each function in vivo is unclear [21].

To efficiently convert the crude glycerol into microbial lipid, exogenous l-proline was added to the media to rescue the adverse effects of crude glycerol stress on the yeast strain. The effect of different l-proline and crude glycerol concentrations were evaluated on lipid production. Lipid were produced by *Rhodosporidium toruloides* AS 2.1389 in a two-stage culture mode under maintained media pH 5.5. Indeed, l-proline improved cell growth, lipid accumulation and lipid yield along with rapid glycerol consumption. Our finding concluded that exogenous l-proline improved overall lipid production efficiency of the strain.

## Results and discussion

### Time course of l-proline uptake, glycerol consumption and lipid production

The culture process was monitored to evaluate the effects of l-proline on the kinetics of lipid production in comparison to that with control. l-proline uptake was followed by instant analysis with IC until l-proline exhaustion.

**Fig. 1:**
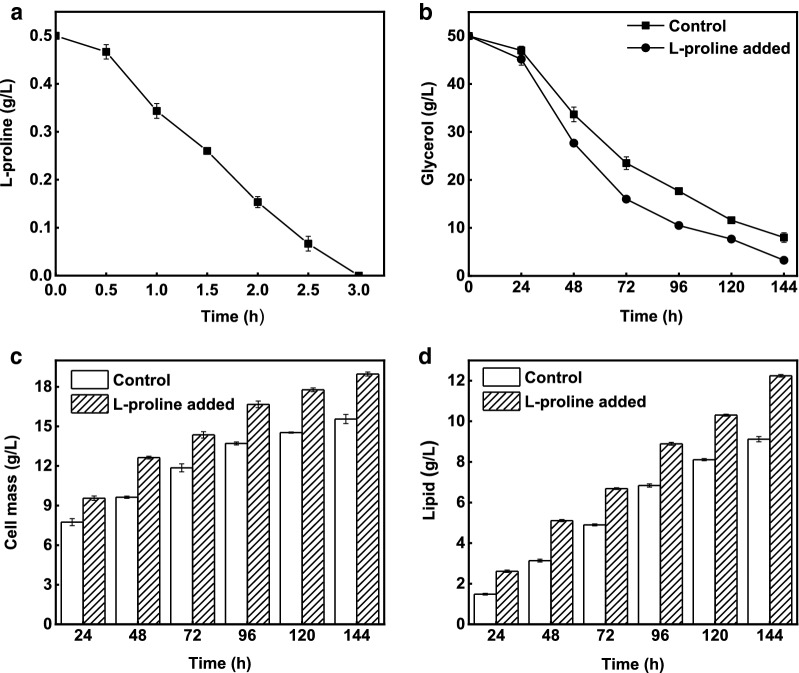
Results of lipid production time course on crude glycerol. **a** l-proline uptake. **b** Glycerol consumption. **c** Cell mass production. **d** Lipid production. Culture was performed at 30 °C and 200 rpm for 144 h

Result indicated a quick gradual decrease in l-proline uptake, and the complete exhaustion occurred within the first 3 h of fermentation (Fig. 1a). The rapid l-proline uptake might was due to the initial stress conditions exerted by the crude glycerol which was indicated by lower growth on control, as well as least glycerol consumption in both media during initial 24 h. However, a speedy glycerol consumption was observed after 24 h, especially on l-proline added media (Fig. 1b). The same phenomena of glycerol consumption was reported previously [31]. Since, l-proline supported higher growth and lipid production throughout the fermentation. The cell mass of 18.9 g/L, lipid 12.2 g/L and lipid content of 64.5% on l-proline added media were all significantly (*P* < 0.001) higher compared to those of control, i.e., 15.5 g/L, 9.1 g/L and 58.6%, respectively (Fig. 1b-d).

Moreover, l-proline not only supported higher growth and lipid production, but also resulted in a highest lipid yield. Along with the higher glycerol consumption of 46.7 g/L, the lipid yield of 0.26 g/g was apparently higher on l-proline added media than that of control i.e., 0.21 g/g. As per our knowledge, the achieved lipid yield was the highest among previous researches with crude glycerol as a substrate (Table 1). It is noteworthy that l-proline addition increased the yeast efficiency by 18%, 25% and 16% in terms of cell mass, lipid and lipid yield, respectively. This demonstrated l-proline as a promising anti-stress agent for *R. toruloides*.

**Table 1:** Lipid production by oleaginous yeasts on glycerol as a carbon source

Strain	Cell mass (g/L)	Lipid (g/L)	Lipid content (%)	Lipid yield (g/g)	References
*R. toruloides* ATCC 10788	08.0	02.5	31.7	0.07	[44]
*R. toruloides* AS2.1389	19.2	09.2	47.7	0.14	[52]
*R. glutinis* TISTR 5159	08.1	04.3	52.9	0.06	[53]
*T. cutaneum*	17.4	05.6	32.2	0.17	[54]
*T. fermentans*	16.0	05.2	32.4	0.16	[54]
*R. toruloides* Y4	24.9	12.2	48.9	0.22	[34]
*R. toruloides* NRRL Y-27012	16.7	07.9	47.2	0.17	[55]
*R. toruloides* AS 2.1389	26.5	10.0	38.0	0.20	[56]
*R. toruloides* AS 2.1389	15.5	09.1	58.6	0.21	This study^a^
*R. toruloides* AS 2.1389	18.9	12.2	64.5	0.26	This study^b^

^*a*^Crude glycerol media without l-proline

^*b*^Crude glycerol media with 0.5 g/L l-proline

### The effects of initial media pH on lipid production

Culture pH has significant effects on cell growth and product accumulation [32]. As H_2_SO_4_ and KOH (or NaOH) are commonly utilized for biodiesel preparation, the by-product crude glycerol may have different pHs, which could be a problem to explore crude glycerol as a substrate for microbial conversion. Therefore, the effects of different initial pH 5.0, 5.5 and 6.0, respectively, were tested on *R. toruloides* lipid production in the presence of 0.5 g/L l-proline, where the control had no l-proline. Note that the yeast initially produce organic acids [33], that might take the culture pH to lower ranges [34]. For the reason, the media was supplied with 100 mM MES buffer to maintain the initially adjusted pH.

**Fig. 2:**
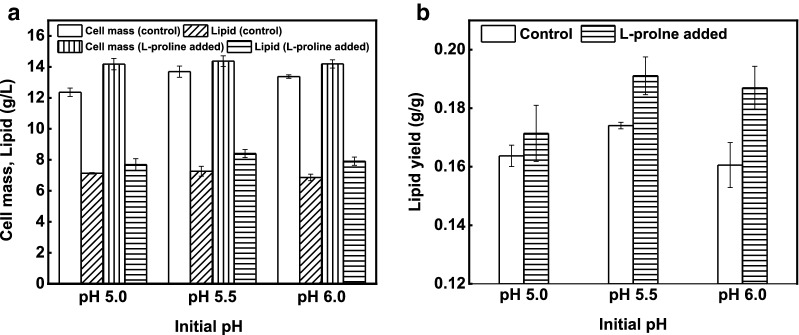
Effects of initial pH on lipid production. **a** Cell mass and lipid production. **b** Lipid yield

Likely, the strain showed lower values on the control at all tested pH ranges. Although significantly lower (*P* < 0.05) cell mass and lipid were achieved on the control at pH 6.0 compared to those of l-proline added media, the lipid and lipid content of 7.2 g/L and 53%, respectively, with highest cell mass of 13.7 g/L were relatively higher at initial pH 5.5 among all controls (Fig. 2a). In contrast, there were no substantial differences in terms of cell mass and lipid titer on l-proline added media at all pH ranges, but the initial pH 5.5 improved overall efficiency of the strain both on control and l-proline added media which was in line with our previous report [34]. At initial pH 5.5, the cell mass reached the highest 14.3 g/L along with 8.4 g/L lipid and lipid contents of 58%, with l-proline addition (Fig. 2a). Moreover, in addition with higher glycerol consumption, the lipid yield of 0.18 g/g was the highest on l-proline added media at initial pH 5.5 (Fig. 2b).

Although there were no substantial differences in lipid production at all applied pH ranges, but higher lipid titer were achieved on all l-proline added media. These results suggested that the *R. toruloides* strain CGMCC 2.1389 has good capability to work on pH ranges between 5.0 and 6.0.

### Effects of methanol and crude glycerol concentration on lipid production

Methanol in crude glycerol comes from a transesterification reaction; however, depends on the separation process, varied concentration of methanol can be found in the aqueous phase [35]. As per previous reports, high contents of methanol in crude glycerol could inhibit cellular growth [36], and lipid production [37]. Therefore, methanol effects were tested on the yeast growth and lipid production in the presence of l-proline. The lipid production media was sterilized at 121 °C for 20 min together with 5 g/L methanol (G + MeOH, GP + MeOH: where “G” stand for control and “GP” stand for media with 0.5 g/L l-proline), or the same amount of methanol was added to the media with syringe filters after sterilization (G–MeOH, GP–MeOH). Where “G ±” stand for control and “GP ±” stand for media with 0.5 g/L l-proline. As methanol has about 64.7 °C boiling point, the sterilized media will have no methanol due to evaporation [38, 39].

**Fig. 3:**
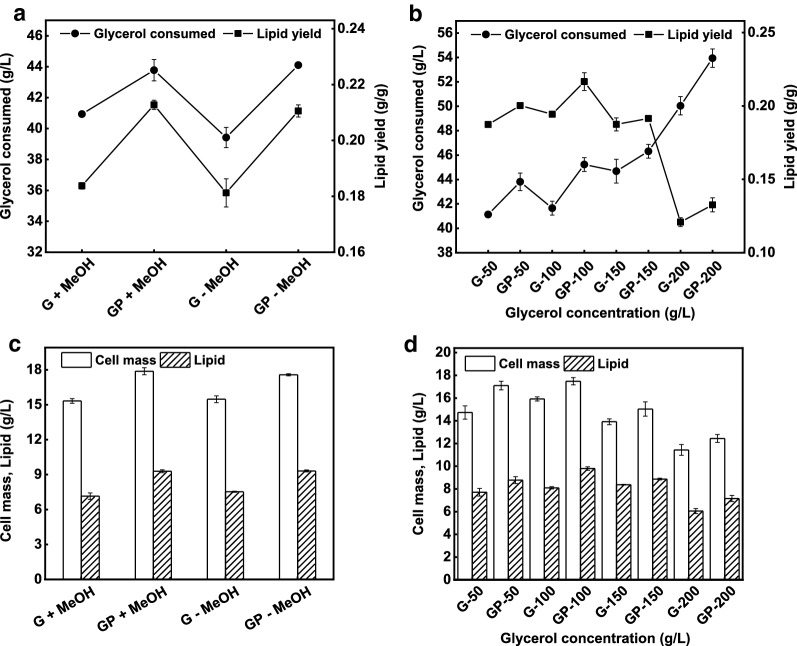
Effects of methanol and crude glycerol on lipid production. **a** Glycerol consumption and lipid yield on glycerol media with and without methanol. **b** Glycerol consumption and lipid yield on different crude glycerol concentration. **c** Lipid production on crude glycerol media with and without methanol. **d** Lipid production on media with different crude glycerol concentration

Later on, no considerable differences were found in cell mass and lipid production in terms of methanol availability. The media supplemented with methanol after sterilization was with slightly higher growth and lipid production, which indicated that 5 g/L methanol had no adverse effects on *R. toruloides* growth and lipid accumulation, but conversely improved the yeast efficiency. However, higher cell mass and lipid were achieved on l-proline added media in both conditions (GP + MeOH, GP–MeOH) compared to that with controls (G + MeOH, G–MeOH) (Fig. 3c). The 15.3 g/L cell mass and 7.0 g/L lipid were achieved on control while these were 17.8 g/L and 9.2 g/L, respectively, on l-proline added media sterilized together with methanol. Hence, the control supplemented with methanol after sterilization was with 15.4 g/L cell mass and 7.5 g/L lipid compared those with 17.5 g/L and 9.3 g/L on l-proline added media. In both conditions, the glycerol consumption was about 40 g/L on both controls with the same lipid yields of about 0.18 g/g. Likewise, the same phenomena was observed with l-proline addition where the glycerol consumption was above 46 g/L and lipid yield of more than 0.21 g/g (Fig. 3a). l-proline addition enhanced cell growth, lipid production, glycerol consumption, and lipid yield compared with control. Moreover, these results suggested that 5 g/L (10% w/w) methanol had no inhibitory effects on *R. toruloides* growth and lipid accumulation [35, 39], but further encouraged the yeast efficiency [34].

Since the high substrate concentration has adverse effects on yeast growth and lipid production due to high osmotic stress [14]. To further study the effects of wide ranges of methanol, salt and glycerol concentration on yeast efficiency in the presence of 0.5 g/L exogenous l-proline, different glycerol concentrations; 50 g/L, 100 g/L, 150 g/L, and 200 g/L were used in the media. Note that, methanol and K_2_SO_4_ were used at 10% w/w each, therefore, with the increase in initial glycerol concentration, the concentrations of salt and methanol were also increased in the media.

Indeed, when the glycerol concentration exceeded 100 g/L, cell growth and lipid accumulation were affected, but compared to that with the control, higher cell mass and lipid were achieved on l-proline added media (Fig. 3d). Since, control with 50 g/L glycerol resulted in 14.7 g/L cell mass, 7.7 g/L lipid and 0.18 g/g lipid yield, whereas, these were 17.1 g/L, 8.7 g/L, and 0.20 g/g, respectively, on l-proline added media (Fig. 3b, d). However, the highest cell mass of 17.4 g/L and lipid of 9.8 g/L were achieved on l-proline added media with 100 g/L crude glycerol, while these were 15.9 g/L, 8.1 g/L, respectively on control. Indicating that 100 g/L glycerol favored the yeast efficiency, where the lipid yield of 0.21 g/g also reached the higher with l-proline addition compared to that with the control i.e., 0.19 g/g, respectively, (Fig. 3b). On the other hand, when the glycerol concentration further increased to 150 g/L, the cell mass and lipid were decreased. The control was with a cell mass of 13.8 g/L, lipid 8.3 g/L, with a lower lipid yield of 0.18 g/g while these were higher on l-proline added media i.e., 15 g/L, 8.8 g/L and 0.19 g/g, respectively. The decrease in cellular growth and lipid accumulation might was due to various factors; the media with the high amount of crude glycerol contained high amount of salt and methanol, which put adverse effects on cell growth and lipid accumulation [40]. It has been known that methanol could have some weak effects on microbial cell membrane fluidity, and thus methanol at 6.5 g/L or higher concentrations inhibited cell growth [35, 41, 42]. Methanol has also been shown to inhibit docosahexaenoic acid production from crude glycerol by *Schizochytrium limacinum* [37]. Similarly, excess salts also generate osmotic pressures that have adverse effects on microbial cell growth [43] and product yield [44]. Likewise, high crude glycerol concentration in the media exerts high osmotic stress [14]. Moreover, the high glycerol concentration might lead to in low oxygen mixing due to high viscosity which resulted lower growth and lipid production, which is also indicated by further decrease in cell mass and lipid production when glycerol concentration increased to 200 g/L (Fig. 3b, d).

These results indicated that below 100 g/L crude glycerol was an optimum concentration for the yeast growth and lipid accumulation (Table 2, entry 3–8). However, the yeast showed higher growth and lipid production efficiency on l-proline added media which proves l-proline as a potential osmoprotectant [45, 46].

**Table 2:** Fatty acids profile of the lipid produced on different glycerol concentrations

Entry	Concentration (g/L)					(g/g)	Relative fatty acid content (% w/w)					
	Glycerol	l-proline	Cell mass	Lipid	Glycerol consumed	Lipid yield	C14:0	C16:0	C16:1	C18:0	C18:1	C18:2
1	50	–	15.5 ± 0.3	9.1 ± 0.1	42.0 ± 1.0	0.217 ± 0.008	2.7 ± 0.1	50.4 ± 0.2	0.9 ± 0.0	14.5 ± 0.2	27.2 ± 0.4	2.3 ± 0.1
2	50	0.5	18.9 ± 0.1	12.2 ± 0.0	46.7 ± 0.0	0.261 ± 0.001	3.0 ± 0.0	55.5 ± 2.0	1.0 ± 0.0	13.5 ± 0.3	23.2 ± 2.1	1.4 ± 0.5
3	100	–	15.9 ± 0.1	8.1 ± 0.0	41.6 ± 0.5	0.194 ± 0.001	2.7 ± 0.9	43.9 ± 3.2	1.3 ± 0.2	11.8 ± 1.0	35.1 ± 3.7	4.5 ± 0.2
4	100	0.5	17.4 ± 0.3	9.8 ± 0.1	45.2 ± 0.5	0.216 ± 0.006	2.6 ± 0.1	48.0 ± 0.8	1.8 ± 0.1	11.2 ± 0.1	34.0 ± 0.8	1.5 ± 0.1
5	150	–	13.9 ± 0.2	8.3 ± 0.0	44.6 ± 0.9	0.187 ± 0.004	2.8 ± 0.1	48.7 ± 0.9	1.1 ± 0.1	13.8 ± 0.3	31.3 ± 1.0	1.4 ± 0.0
6	150	0.5	15.0 ± 0.6	8.8 ± 0.0	46.3 ± 0.5	0.191 ± 0.000	2.8 ± 0.2	47.2 ± 0.7	1.9 ± 0.1	10.0 ± 0.2	33.7 ± 0.6	3.8 ± 0.4
7	200	–	11.4 ± 0.4	6.0 ± 0.2	50.0 ± 0.7	0.120 ± 0.003	2.6 ± 0.1	48.2 ± 0.9	1.1 ± 0.2	13.5 ± 0.2	32.0 ± 1.3	1.7 ± 0.0
8	200	0.5	12.4 ± 0.3	7.1 ± 0.2	53.9 ± 0.7	0.132 ± 0.004	2.5 ± 0.0	46.2 ± 0.1	1.8 ± 0.0	10.2 ± 0.2	35.3 ± 0.1	3.4 ± 0.1

### Effects of l-proline level and supply time on lipid production

Previous reports confirmed improved yeast tolerance to various inhibitors by enhancing l-proline biosynthesis [45, 46]. However, the excess of l-proline accumulation in the cytosol might also be toxic to yeast cells [47]. In order to find an optimum l-proline concentration, the media was supplied with different levels of l-proline g/L; 0.0 (control), 0.25, 0.5, 0.725, 1.0, and 1.25.

**Fig. 4:**
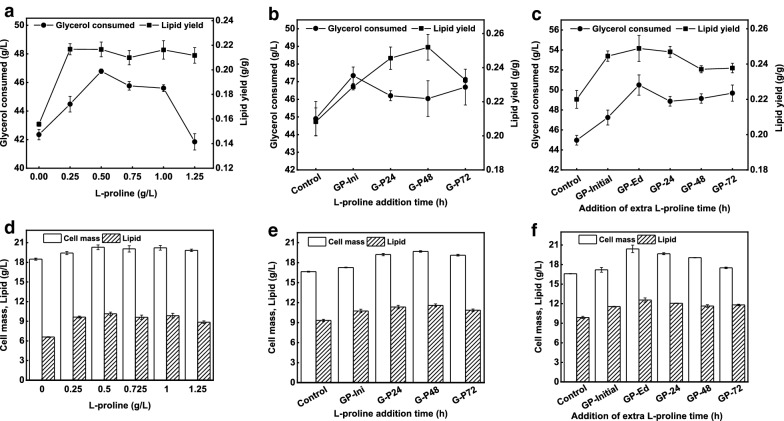
Effects of l-proline on lipid production from crude glycerol. **a** Effects of different l-proline levels on glycerol consumption and lipid yield. **b** Effects of 0.5 g/L l-proline addition after different time intervals on glycerol consumption and lipid yield. **c** Effects of 0.5 g/L additional l-proline supply after different time intervals on glycerol consumption and lipid yield. **d** Effects of different l-proline levels on lipid production. **e** Effects of 0.5 g/L l-proline addition after different time intervals on lipid production. **f** Effects of 0.5 g/L additional l-proline supply after different time intervals on lipid production

In fact, l-proline at all tested levels improved overall fermentation efficiency of the strain in terms of glycerol consumption, growth, lipid production and yield. The cell mass of 20.3 g/L, lipid of 10.1 g/L and lipid contents of 49% were significantly higher (*P* < 0.001) at 0.5 g/L l-proline addition than those of 18.4, 6.6 and 35%, respectively, on control. Furthermore, the lipid yield of 0.21 g/g was also far higher with 0.5 g/L l-proline addition than that of control i.e., 0.15 g/g. These accounted for 34% and 28% increase in lipid and lipid yield, respectively, (Fig. 4d). Likewise, 0.5 g/L l-proline addition also improved the glycerol consumption of i.e., 46.7 g/L (Fig. 4a). Although there were no substantial differences in lipid contents and yield among all l-proline concentrations used, but the lipid contents of 44% with glycerol consumption of 41.8 g/L were comparatively the lowest with 1.25 g/L l-proline addition, indicating that the proline concentration was a bit higher which might affected the yeast efficiency.

To find a suitable l-proline supply time, 100 g/L crude glycerol media with 10% methanol and K_2_SO_4_, respectively, was supplied with 0.5 g/L l-proline at different time intervals: initially (GP-Initial), at 24 h (G-P24), 48 h (G-P48) and 72 h (G-P72), respectively (Fig. 4b, e). The control carried no l-proline. After 144 h, significantly higher (*P* < 0.001) cell mass and lipid were achieved with l-proline addition compared with the control. The highest cell mass of 19.7 g/L and lipid of 11.6 g/L attained at G-P48 followed by G-P24, where these were 19.2 g/L, 11.3 g/L, respectively. Moreover, G-P72 was with a bit lower lipid of 10.8 g/L, which might was due to late l-proline supply, where the yeast took less advantage of l-proline (Fig. 4e). Even though the differences were not substantial with 0.5 g/L l-proline addition after different time intervals, but the cell mass and lipid were higher than those of control and GP-Initial. Since, the glycerol consumption and lipid yield reached the highest i.e., 47.3 g/L and 25.1, respectively, on G-P48. In contrast, the control was with 44.9 g/L glycerol consumption and a lower lipid yield of 20.8 (Fig. 4b).

We further investigated the effects l-proline on lipid accumulation, the same media as above with 0.5 g/L initial l-proline contents was additionally supplemented with 0.5 g/L l-proline after different time intervals, i.e., 24 h (GP-24), 48 h (GP-48), and 72 h (GP-72). Unlikely, one set was supplied additionally with 0.125 g/L l-proline every 24 h interval for 4 days (GP-Ed). The control had no l-proline, while one set initially supplemented with 0.5 g/L l-proline was kept without additional l-proline supply (GP) (Fig. 4f).

After 144 h, significantly higher (*P* < 0.001) cell mass and lipid were achieved on all l-proline added media compared to those with the control. Although, compared with GP, the cell mass and lipid were higher at all media with additional l-proline supply. Since, GP-Ed was with highest cell mass of 20.4 g/L and 12.5 g/L lipid which indicated that the readily available l-proline had more encouraging effects on the yeast efficiency. Moreover, GP-24 was also likely with higher cell mass of 19.6 g/L and lipid of 12.0 g/L. On the other hand, the cell mass and lipid were bit lower i.e., 17.2 g/L and 11.5 g/L, respectively, on GP-Initial while the control was with the lowest cell mass of 16.6 g/L and lipid of 9.9 g/L (Fig. 4f). Similarly, the lipid yield reached the highest 0.24 g/g with 50.5 g/L glycerol consumption on GP-Ed (Fig. 4c). In fact, additional l-proline showed more encouraging effects on the cell growth and lipid accumulation.

All these results demonstrated that exogenous l-proline not only improved the lipid production but also relieved the stress conditions and promoted the cell growth [45, 46]. Though, the additional l-proline showed more promising effects compared with only initial l-proline supply which indicates low amount of l-proline addition after intervals might be prove more beneficial.

The results of microbial lipid production on crude glycerol by different oleaginous yeasts are summarised in Table 1. Our work demonstrated comparatively improved results in terms of lipid titer and yield, which demonstrated l-proline as a potential anti-stress agent. However, engineering *R. toruloides* for improved l-proline biosynthesis could contribute to biorefinery with enhance lipid titer, yield and short production time by quick resource utilization.

### Overview on l-proline metabolism in yeast

The yeasts cell biosynthesize l-proline in the cytoplasm from glutamate through γ-glutamyl kinase, γ-glutamyl phosphate reductase, and Δ^1^-pyrroline-5-carboxylate reductase produced by the genes PRO1, PRO2 and PRO3, respectively [48, 49]. Moreover, *S. cerevisiae* additionally biosynthesize l-proline by converting arginine with arginase and ornithine aminotransaminase produced by CAR1 and CAR2 genes, respectively [50]. On the contrary, l-proline is catabolized to glutamate by proline oxidase and Δ^1^-pyrroline-5-carboxylate dehydrogenase produced by the genes PUT1 and PUT2, respectively, [51]. The l-proline metabolic pathway in yeast is described in Additional file 1: Figure S1).

### Fatty acid compositional profiles of the lipid products

The majority of lipids produced by oleaginous yeasts are triacylglycerols (TAG), which mainly consist of long chain fatty acids including C14:0 (myristic acid), C16:0 (palmitic acid), C18:0 (stearic acid), C18:1 (oleic acid) and C18:2 (linoleic acid). The fatty acid profiles may vary to some extent depending on the culture media and cultivation conditions [57]. The fatty acid methyl esters (FAME) of the transmethylated lipid samples were analysed with GC-FID. Myristic acid, palmitic acid, stearic acid and oleic acid were found in all samples. However, the contents of those with 16 and 18 carbon atoms were above 96% of the total fatty acids, where palmitic acid accounted for about 50% followed by oleic acid (Table 2). It is noteworthy that oleaginous yeasts produce lipids with 16 and 18 carbon containing fatty acids as the major fractions [58], which are considered as a potential feedstock for biodiesel industry [59].

## Conclusion

We have illustrated the effects of exogenous l-proline on the red yeast *R. toruloides* lipid production efficiency under crude glycerol stress. l-Proline improved the yeast growth and lipid production on glycerol in the presence of high amount of methanol and salt. Our finding concluded that exogenous l-proline not only improved the cell growth and lipid production by 22% and 34%, respectively, but also enhanced the lipid yield by 20% along with higher glycerol consumption. In conclusion, engineering *R. toruloides* for enhanced endogenous l-proline synthesis can aid in fermentation processes with a speedy resource utilization and efficient lipid production under several environmental anxieties.

## Materials and methods

### Microorganism, media and growth conditions

The red yeast strain *R. toruloides* CGMCC 2.1389 was obtained from China General Microbiological Culture Collection Centre. The strain was maintained at 4 °C on yeast extract-peptone-dextrose (YEPD) agar plate containing 20 g/L glucose·H_2_O, 10 g/L peptone, 10 g/L yeast extract and 20 g/L agar, and sub-cultured twice a month. Peptone (total nitrogen 14.5% and phosphorus 0.14%) and yeast extract (total nitrogen 9% and phosphorus 1.3%) were obtained from Aoboxing Biotech. Co. Ltd. (Beijing, China). The seed cells were cultured on YEPD medium containing 20 g/L glucose·H_2_O, 10 g/L yeast extract, and 10 g/L peptone, pH 6.0.

The composition of crude glycerol varies but typically comprises of glycerol (60–90%), salts (5–6%) and methanol (4–6%) [14] [15] [52]. In this work, synthetic crude glycerol stock solution was prepared by dissolving pure glycerol, methanol and K_2_SO_4_, in distilled water to final concentrations of 60 wt %, 6 wt % and 6 wt %, respectively [34]. Therefore, the lipid production media contained (unless otherwise specified) glycerol 50 g/L, methanol 5 g/L, K_2_SO_4_ 5 g/L, and l-proline 0.5 g/L, where the control contained no l-proline. The initially set media pH 5.5 with HCl or KOH solutions was maintained with 100 mM 2-(N-morpholino)ethanesulfonic acid (MES) buffer. All of the culture media were sterilized at 121 °C for 20 min. Note that, methanol was supplied to the lipid production media after sterilization (unless otherwise specified).

### Seed culture inoculation

Two-stage culture conditions were employed with a high initial cells density. Shortly, *R. toruloides* CGMCC 2.1839 cells were inoculated in YEPD media and cultivated at 30 °C, 200 rpm for 40 h. Cells from 40 mL of preculture were then collected at 5000 rpm centrifugation for 5 min, washed twice with deionized water and used as seed for lipid production.

The seed cells at an initial concentration of 7.0 g/L were then transferred into 250 mL flask containing 50 mL of lipid production media, and incubated at 30 °C, 200 rpm for 120 h (unless otherwise specified). All culture experiments were performed in triplicates.

### Analytical methods

After incubation, cells were harvested at 8000 rpm centrifugation for 5 min, washed twice with deionized water and dried at 105 °C to constant weight [60]. The cell mass was measured gravimetrically, the cell mass was expressed in g/L.

To extract lipid, the dried cells were digested with 4 M HCl solution at 78 °C for 1 h at 200 rpm shaking, extracted via methanol/chloroform (1:2, v/v). The extracts were washed with 0.1% NaCl and passed through anhydrous Na_2_SO_4_ pad, then concentrated under reduced pressure,  dried at 105 °C to constant weight [61], and the lipid was measured gravimetrically. The total produced lipid were expressed in g/L, the cellular lipid contents in percent (%) were calculated as gram lipid produced per gram cell mass. The lipid yield was defined as gram lipid produced per gram glycerol consumed (g/g glycerol consumed).

The fatty acids compositional profile of the transesterified lipid samples were analysed with gas chromatography (GC) according to a previous work [62]. Briefly, the lipid sample (70 mg) was treated with 0.5 mL of 5% KOH methanol at 65 °C for 50 min, then 0.2 mL BF_3_ diethyletherate and 0.5 mL methanol were added. The mixture was refluxed for 10 min, cooled, and extracted with n-hexane. The organic layer was washed twice with distilled water and used for fatty acid compositional analysis. Then, the compositional profiling of fatty acid was measured by a 7890F GC (Techcomp Scientific Instrument Co. Ltd., Shanghai, China), which was equipped with a cross-linked capillary FFAP column (30 m × 0.25 mm × 0.25 mm) and a flame ionization detector. The flow rates for N_2_, H_2_, and air were 720 mL/min, 30 mL/min, and 100 mL/min, respectively. The injection port, oven, and detector temperature were set at 250 °C, 190 °C and 280 °C, respectively. The injection volume was 0.5 µL. Fatty acids were identified and quantified by comparing the retention time of those with standards and the respective peak areas and area normalization.

l-proline and glycerol analysis were performed with ion chromatography (IC) by using an ICS-5000 series instrument (Thermo-Fisher Scientific Waltham, MA, USA). The AminoPac PA10 column set consisting of a guard column (4 mm × 50 mm) and an analytical column (4 mm × 250 mm). Gradient elution was performed at a flow-rate of 0.25 mL/min, with water, sodium hydroxide, and sodium acetate mobile phases using the ternary gradient method. The temperature of the column was maintained at 30 °C. Results were quantified according to the l-proline and glycerol standard chromatogram. Additionally, glycerol was also quantified with a previously established spectrophotometric procedure by combining the Malaprade reaction and the Hantzsch reaction [63].

### Statistical analysis

The jamovi project (2020) jamovi (Version 1.2) [Computer Software] was used for statistical analysis. One-way ANOVA was conducted to compare control group with l-proline added group. Data with *p* < 0.05 was considered statistically significant.

## Supplementary information

**Additional file 1: Figure S1.** l-proline metabolic pathway in yeast.

## Data Availability

Additional file 1 contains supporting information.

## Abbreviations

CGMCC: China General Microbiological Culture Collection Centre
YEPD: Yeast extract-peptone-dextrose
MES: 2-(N-morpholino)ethanesulfonic acid
GC: Gas chromatography
IC: Ion chromatography
HCl: Hydrochloric acid
KOH: Potassium hydroxide
FAMEs: Fatty acid methyl esters
