# Supplementary material for: Exogenous l-proline improved Rhodosporidium toruloides lipid production on crude glycerol
Source: Biotechnol Biofuels. 2020 Sep 14;13:159. doi: 10.1186/s13068-020-01798-6 (PMC7490893; doi:10.1186/s13068-020-01798-6)
Supplement: Supplementary file 1 — Additional file 1: Figure S1. l-proline metabolic pathway in yeast. [file 13068_2020_1798_MOESM1_ESM.docx]

**Supplementary Information**

The additional file 1 should be placed in supplementary information’s.

**Additional file 1**

**
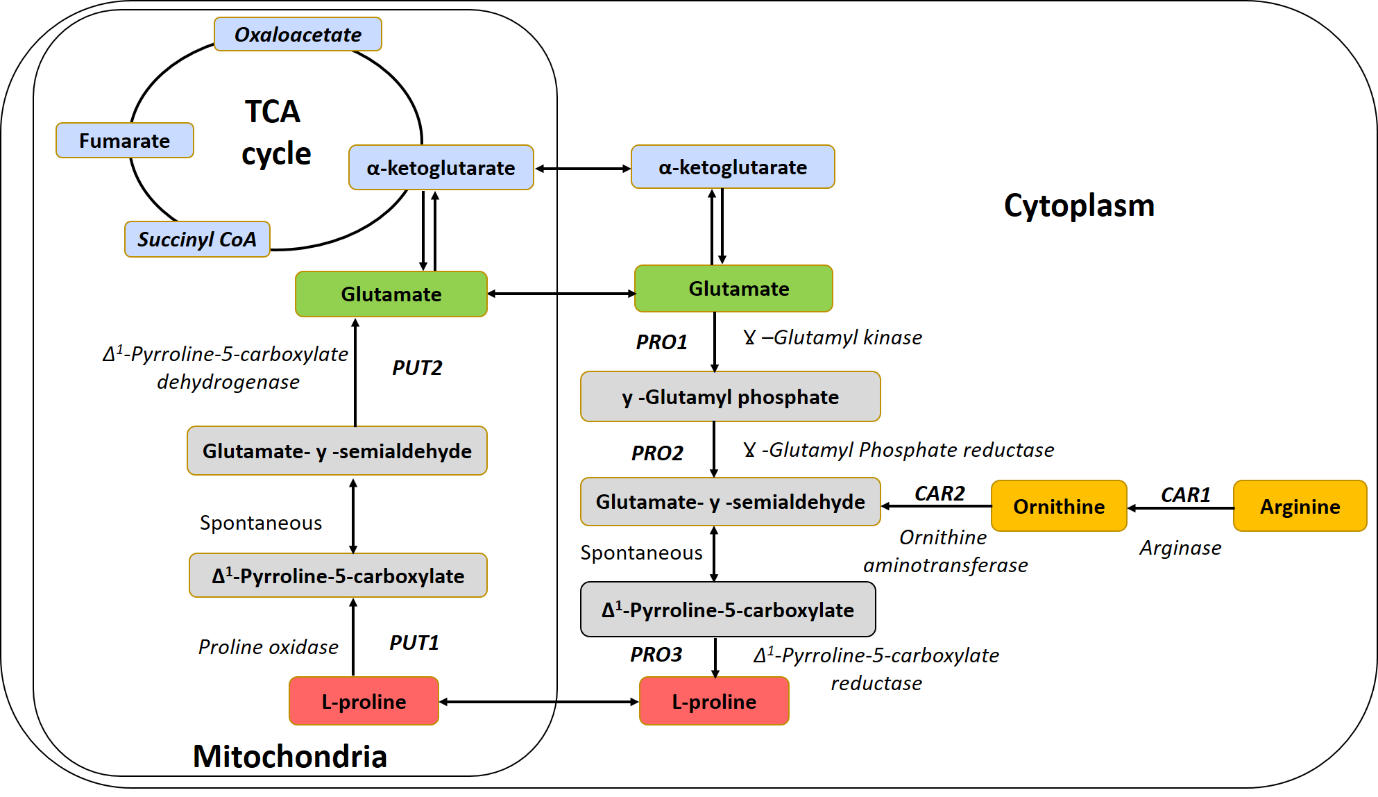
**

**Fig. S1** L-proline metabolic pathway in yeast.
